# Analysis of Interface Fusion Effect between Old and New Asphalt under Plant Mixing and Cold Recycling Mode Based on Molecular Dynamics Simulation

**DOI:** 10.3390/ma14164637

**Published:** 2021-08-18

**Authors:** Peilei Zhou, Wensheng Wang, Zhe Yu

**Affiliations:** 1 College of Transportation, Jilin University, Changchun 130025, China; zhoupeilei@jlu.edu.cn; 2 College of Construction Engineering, Jilin University, Changchun 130026, China; 3 School of Public Art, Changchun University of Architecture and Civil Engineering, Changchun 130000, China

**Keywords:** molecular dynamics, cold recycling, diffusion coefficient, interfacial fusion degree, rejuvenator

## Abstract

Road construction consumes a lot of resources and produces a lot of waste and other pollutants. With the emergence of a resource and energy crisis, how to make efficient use of rap has become the research focus of scientific researchers. The interface fusion effect of old and new asphalt in plant mixing and cooling recycling mode is analyzed in order to improve the utilization rate of old asphalt in reclaimed asphalt pavement. In this paper, Materials Studio software was used to establish a bitumen model using the method of four components of bitumen, and then the rationality of the model was verified by density, solubility number and atomic radial distribution function, and the diffusion coefficient obtained from the mean square displacement (*MSD*) was taken as its evaluation index. The results showed that the diffusion model tends to be stable after 20 ps, and the degree of diffusion increases with the increase in temperature. The degree of diffusion of new asphalt to old asphalt and the degree of diffusion of old asphalt to new asphalt are basically very similar; however, there are some differences at different temperatures. Only a small part of the surface contact between old and new asphalt has been fused, which accords with the partial fusion theory. Compared with Panjin 90# asphalt, the diffusion coefficient of Zhonghaiyou asphalt increases faster with the increase in temperature. The diffusion coefficient increases by 64.3% with the increase of the content of rejuvenators after adding different rejuvenators into the new asphalt. Clarifying the interface fusion effect will be helpful to guide the optimization design of cold-mixing recycled asphalt mixture more scientifically and reasonably. Future research should focus on increasing the fusion effect of old and new asphalt, and explore its influence on the conventional road performance of asphalt mixture.

## 1. Introduction

China’s highway traffic has developed very rapidly since the steady implementation of the 13th Five-Year Plan [1,2]. By the end of 2018, the total mileage of national roads was 4.86 million km, of which expressways were 142,000 km [3,4]. However, most of the asphalt pavement in China will need to be maintained and repaired with the increase in service time, and tens of millions of tons of waste asphalt mixture will be generated in highway reconstruction and renovation projects every year [5,6,7]. It is necessary to pay attention to the recycling of waste asphalt mixture due to the increasing shortage of petroleum, asphalt, and other non-renewable resources [8,9,10]. At present, China’s pavement rejuvenation technology can be divided into thermal rejuvenation and cold rejuvenation [11,12,13,14]. Compared to cold rejuvenation, thermal rejuvenation technology will cause a large amount of pollution, which is not conducive to energy conservation [15,16]. The plant mixed cold rejuvenation is more convenient than local cold rejuvenation, so the technology has a very objective development prospect [17,18,19,20]. In the process of cold asphalt rejuvenation, the new asphalt and rejuvenator are mixed with the old asphalt on the surface of the reclaimed asphalt pavement (RAP) under certain temperature and stirring conditions, and then diffused and miscible with it from outside to inside. However, the effect depth of this process is limited, and the old asphalt coated inside RAP does not participate in the rejuvenation [21]. At present, most of the domestic cold-mixing recycling technologies only use RAP as a “black aggregate”, which not only fails to make full use of the value of old asphalt in RPA, but also leads to different thickness of asphalt film on new and old aggregates, thus affecting the performance of asphalt mixture [22,23,24]. At present, there is a lack of evaluation index for the diffusion degree of new and old asphalt, so it is necessary to find a reasonable evaluation index.

At present, the rejuvenation technology of RAP in foreign countries has been relatively mature, especially in the United States and Japan, where they are improving the utilization rate of old asphalt; asphalt material aging and rejuvenation mechanism research is more mature [24,25,26,27]. However, the research on RAP rejuvenation started late in China, but has developed rapidly in recent years [28,29,30]. Studies on diffusion characteristics are mainly divided into macro experiment and microanalysis. Macroscopically, the dynamic shear rheological (DSR) experiment can be used to analyze the significant effects of time, temperature, asphalt variety, and rejuvenator on composite shear modulus by means of variance analysis, so as to compare their effects on diffusion [31,32,33,34]. On the micro level, infrared spectrum analysis, gel chromatography, thin layer chromatography, Fourier infrared spectroscopy and other advanced means are adopted to study asphalt mixture, and molecular simulation technology is used to simulate the diffusion behavior of old and new asphalt [35,36,37,38].

However, the macro experiment is limited in the study of mechanism, so the diffusion behavior of old and new asphalt can be understood more deeply from the perspective of micro level [6,39]. At present, most studies have considered the influence of material grading and asphalt grade on the performance of recycled asphalt mixture from a macroscopic perspective, and proposed corresponding control indexes. However, the existing pavement is prone to early damage for the reason that the interface fusion effect is difficult to be determined in the macroscopic experiment. From the microscale, the asphalt components can achieve a good fusion effect. The asphalt is divided into four components, which would be described in the following sections [7,40]. With the rapid development of computers and the difficulty of carrying out a large number of experiments, molecular simulation technology can easily simulate a lot of experiments, so as to verify the rationality of simulation through macroscopic experiments [41,42].

This paper takes the fusion behavior of old and new asphalt as the research object. In this paper, Material Studio software [43] was used to establish a four-component model of asphalt, and then the rationality of the model was verified through density, dissolved quantity, and atomic radial distribution function. The longer the simulation time ps, the better the effect. Generally, the 200 ps asphalt model can reach a stable state. Then, molecular dynamics simulation of 200 ps was carried out; further, the temperature diffusion effect of the interface between old and new asphalt, asphalt type and recycled asphalt type was studied based on the *MSD* diffusion coefficient. The fusion behavior of old and new asphalt is analyzed from the micro point of view; the micro action mechanism of recycled asphalt mixture is analyzed, and the real state of the diffusion of old and new asphalt is studied, which provides a certain basis and reference for the mix proportion design and construction process optimization of large amounts of plant mixed cold recycled asphalt mixture in the future.

## 2. Molecular Simulation Modeling

### 2.1. Introduction of Molecular Simulation Technique

Molecular dynamics simulation is a commonly used micro simulation method. By constructing a molecular model, we can study the dynamic characteristics of the model under different force fields, different temperatures, and time conditions. At present, many researches on the micro aspects of asphalt mixture performance at home and abroad have adopted molecular dynamics simulation. Different studies have proposed different asphalt model establishment methods—from the most original single molecule model to more than a dozen asphalt models jointly constructed by different molecules. In this paper, Materials Studio software is selected to construct asphalt molecular model with four molecular model.

Molecular dynamics simulation is a technique based on the theory and calculation methods of statistical mechanics, simulating the interaction of molecules under different conditions by computer. The key of molecular simulation technology is to construct the appropriate molecular model, and then select the appropriate force field. Under the action of the force field, the molecular structure and interaction of atoms adjacent to the molecule will generate different potential energy [44].

Molecular dynamics simulation process: Firstly, get the initial coordinates and velocity or followed by a molecular simulation process. Next, calculate the potential function and each particle stress, and then calculate the particle kinematics equation of Newton, applying leapfrog algorithm for particle redistribution new coordinates and speed. Finally, the output characteristics of the thermodynamic system static, such as temperature, energy and stress, if necessary, coordinates and velocities can also be solved [45].

The application of molecular dynamics simulation mainly includes the following three parts: firstly, selection of force field and ensemble; secondly, reasonable establishment of model; thirdly, molecular dynamics simulation and result analysis [46]. In a force field, the atoms in the structure interact with each other, and the appropriate force field should be selected for different molecular models. Compass force field is suitable for polymers, small organic molecules, etc., which is suitable for asphalt binders. Also, since the actual volume of old and new asphalt will change in the diffusion process, isothermal-isobaric thermostat (NPT) ensemble is constant-pressure and constant-temperature. It is proposed to use the NPT ensemble for calculation and analysis [6]. Therefore, the compass force field and NPT ensemble were used in this paper.

### 2.2. Establishment of Asphalt Molecular Model

#### 2.2.1. Materials

The asphalt molecular model was established by assembly method in this paper. Firstly, the molecular representative structure that can represent the four components of the four-component method was determined. Then, four kinds of asphalt samples of Panjin 90# asphalt (Panjin North asphalt Co., Ltd., Panjin, China), Panjin 90# asphalt after long-term aging, Zhonghaiyou 90# asphalt (Zhonghai asphalt Co., Ltd., Binzhou, China), Zhonghaiyou 90# asphalt after long-term aging were established, and the diffusion behavior between asphalt and long-term aging asphalt was studied. After measuring the four-component method and consulting relevant data, the mass percentages of the four components of the two asphalt samples are shown in Table 1.

**Figure 1:**
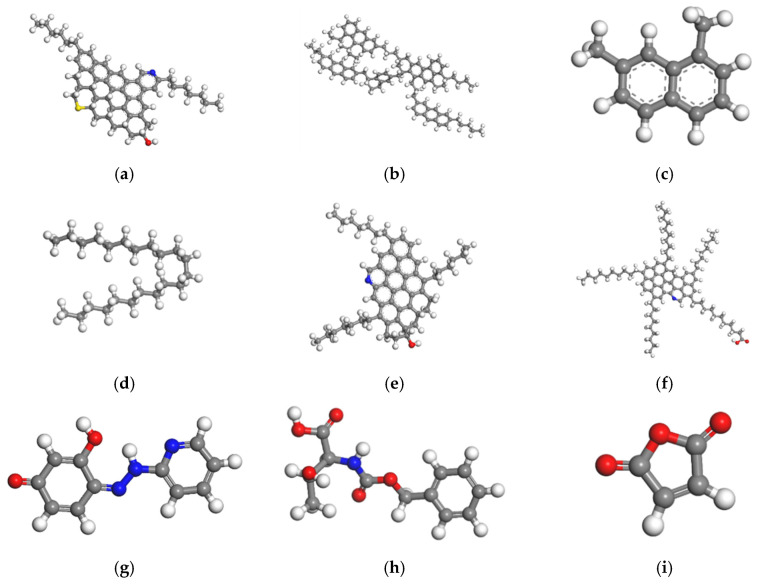
Diagram of the optimized molecular structure of each component: (**a**) Panjin 90# asphaltenes; (**b**) Panjin 90# nasphalthene aromatics; (**c**) Polar aromatics; (**d**) Saturates; (**e**) Zhonghaiyou 90# asphaltenes; (**f**) Zhonghaiyou 90# nasphalthene aromatics; (**g**) Rejuvenator type I; (**h**) Rejuvenator type II; (**i**) Rejuvenator type III.

In general, asphaltenes have complex structural characteristics of aromatic ring with high condensation and macromolecular structure with a small number of other atoms and long branched chains, while asphaltenes produced in different regions also have different asphaltene structural characteristics. Not only that, different results have been obtained in the study of gelatins. Therefore, the actual situation of selected asphalt samples should be considered in the selection of asphaltene and gelatin molecules [47]. Wang et al. simulated the compatibility between asphalt and rubber powder in Panjin 90# asphalt and verified its feasibility, which provides a reference for the component molecular structure of Panjin 90# asphalt [48]. Rejuvenator type I and rejuvenator type II are lightweight components-based materials with a benzene ring structure. Compared to the rejuvenator type II, rejuvenator type I has more benzene ring and shorter branch chain. Rejuvenator type III is maleic anhydride. The representative molecular formulas of each component of asphalt samples are shown schematically in Figure 1.

#### 2.2.2. Molecular Simulation Method

**Figure 2:**
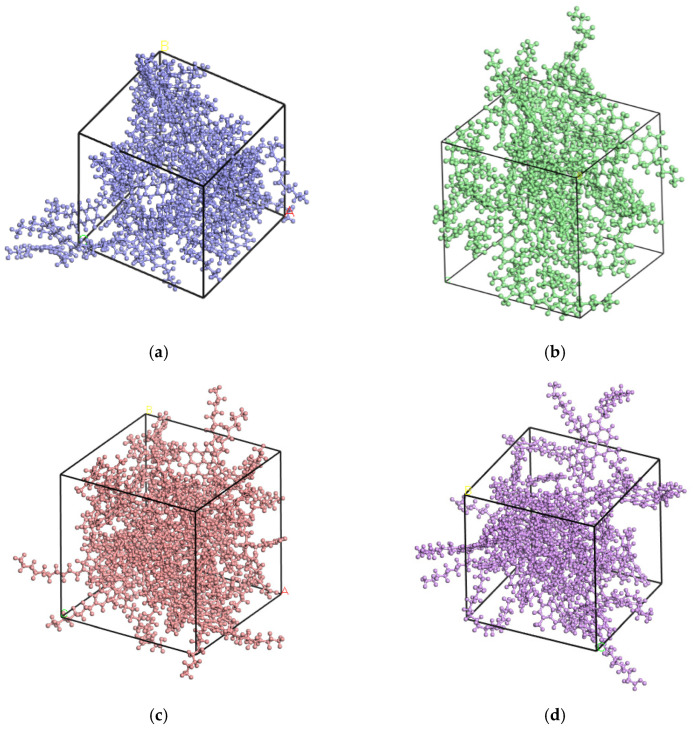
Two types of new asphalt and old asphalt models: (**a**) Panjin 90# asphalt; (**b**) aged Panjin 90# asphalt; (**c**) Zhonghaiyou 90# asphalt, and (**d**) aged Zhonghaiyou 90# asphalt.

According to the proportions of different asphalt components and the molecular weight of the corresponding components, the mole ratio of each component in asphalt molecules was calculated, and the number of molecules was selected. Then the representative molecules were constructed by using the Amorphous Cell module in Materials Studio software (Version 2019, Accelrys Software Inc., San Diego, CA, USA). The dynamic simulation calculation was added due to the instability of initial intramolecular and intermolecular energies in the asphalt bitumen molecular model. However, the incompatibility of structural calculation can lead to failure of the model, requiring the optimization of the asphalt molecular geometry configuration to obtain a more reasonable configuration. It is still necessary to anneal the asphalt molecules to reduce their energy considering the complex molecular structure inside the asphalt. After that, the dynamics simulation calculation of asphalt molecules was carried out. The specific steps are as follows:

- First, the construction in amorphous cell module was used to model the asphalt molecules.
- Second, the geometry optimization was accomplished by the Forcite module.
- Last, the anneal function of the Forcite module was used to backpedal asphalt molecules, in which the initial temperature is 300 K, the intermediate temperature is 500 K, and 25 cycles were carried out; the models are shown in Figure 2.

## 3. Asphalt Model and Diffusion System

### 3.1. Material Parameters

#### 3.1.1. Density

The density of Panjin 90# asphalt calculated by the asphalt molecular model is 0.966 kg/m^3^, and that of Zhonghaiyou 90# asphalt is 0.983 kg/m^3^. Compared with the actual density of asphalt, which is 1.000 kg/m^3^, the error is less than 4%. Therefore, it can be considered that the density of the asphalt molecular model is reasonable.

#### 3.1.2. Solubility Parameter

Redelius proposed the concept of solubility parameter in the study of a polymer system, which can represent the interaction between simple liquids [49]. The quality polarity can be reflected by the solubility parameter of the material, according to which the compatibility between each molecule can be determined. Therefore, the concept of solubility parameter can be used to judge the stability of the asphalt molecular model. Forcite module in Materials Studio software was used to calculate cohesive energy density (CED), and the results are shown in Table 2. The difference of the solubility parameters between the four components of two kinds of asphalt is less than 4 (J/cm^3^)^1/2^, so both of them can be considered to have stable structures.

### 3.2. Atomic Radial Distribution Function

**Figure 3:**
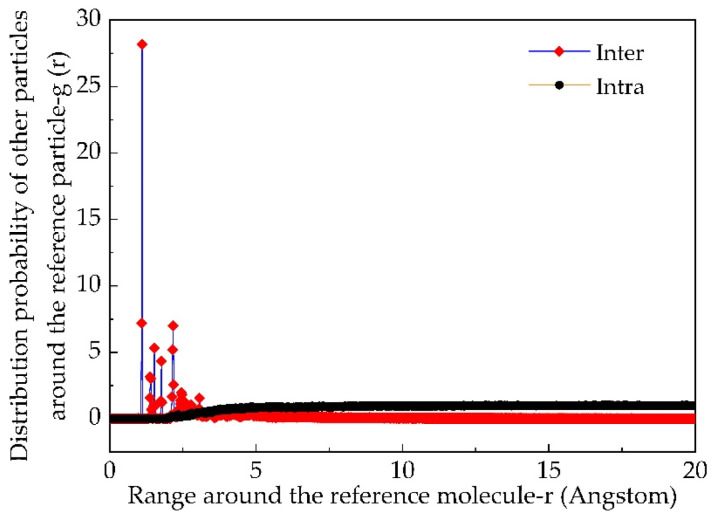
The dependence of atomic radial distribution as a function of the range (r).

Atomic radial distribution function can analyze the distribution law and situation of atoms, so as to judge whether the asphalt model established conforms to the actual situation. The Analysis module in Forcite was used to analyze the atomic radial distribution functions between and within molecules of the asphalt molecular model. Since the results of the two types of new asphalt and aging asphalt are similar, the results of Panjin 90# asphalt are taken as an example, as shown in Figure 3. It shows that the curve of atomic radial distribution function between the molecules in the asphalt molecular model rises slowly and finally converges to 1. However, the atomic radial distribution function within the molecule gradually stabilizes around 0 after the peak [50]. Asphalt is a typical amorphous material, whose atoms in macromolecules show near-range order and distant disorder. Moreover, van der Waals force is the most important intermolecular force, and according to Figure 3, the established asphalt molecular model can be judged to be reasonable [51,52].

### 3.3. Establishment Method of the New-Old Asphalt Diffusion System Model

**Figure 4:**
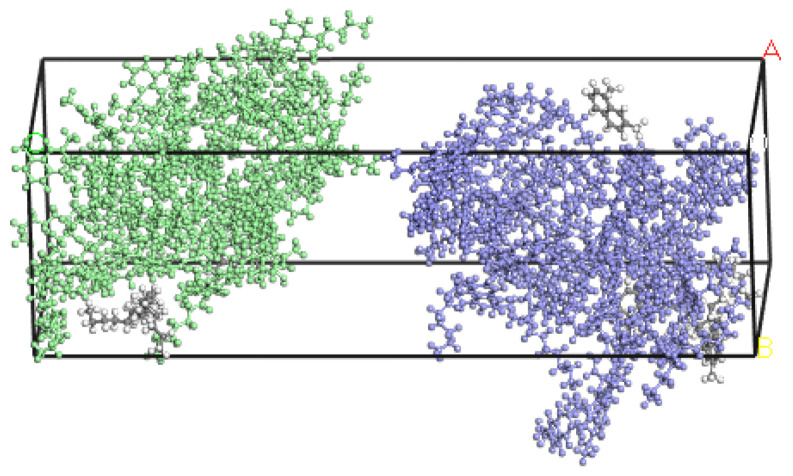
New-old asphalt contact interface model.

The Build Layers command was used to model the new-old asphalt contact interface. Both the new and old asphalt models were established at a temperature of 300 K. Since this paper studied the fusion effect of the interface between the old and the new asphalt in the cold rejuvenation mode, the construction temperature is usually between 10~60 °C. The simulation temperatures were selected as 283.15 K, 293.15 K, 303.15 K, 313.15 K, 323.15 K, and 333.15 K. The dynamic simulation was carried out in Dynamic, and the ensemble was NPT ensemble. The interface model between Panjin 90# asphalt and the long-term aging Panjin 90# asphalt and Zhonghaiyou 90# asphalt and the long-term aging Zhonghaiyou 90# asphalt was simulated with 200 ps, respectively. Taking 303.15 K as an example, the following Figure 4 shows the new-old asphalt contact interface.

### 3.4. Diffusion Evaluation Index of the New-Old Asphalt System

#### 3.4.1. All Azimuth Shift

In the research center of Brownian motion, Einstein found that the sum of squares of the distance traveled by randomly moving particles has a linear relationship with time, that is:

<*r*^2^> = 6*Dt* + *C*, (1)

*MSD* = <|*r*(*t*) − *r*(0)|^2^>, (2)

(3) $$D=\underset{t\to \infty }{\lim}\frac{1}{6t}<|r(t)-r(0)|>,$$

where <*r*^2^> represents the mean azimuth shift, *D* and *C* represent constants, <> represents the mean and r represents the displacement. The diffusion coefficient of the model cannot be directly obtained by molecular simulation. After the data of *MSD* is derived according to the above formula, the slope of the *MSD* is calculated by linear fitting, that is, the mean azimuth shift. The 1/6 of the mean azimuth shift is the diffusion coefficient [53,54].

#### 3.4.2. Diffusion Coefficient

The diffusion coefficient can reflect the diffusion rate of the interface between the old and new asphalt, that is, the faster the diffusion rate is, the better the fusion degree is. Therefore, the fusion degree of the interface between the old and new asphalt can be evaluated. For the diffusion phenomenon in this paper, it is considered that the new old asphalt will diffuse after contacting at a certain temperature, and its diffusion rate is affected by temperature and diffusion time. Since diffusion varies with time and temperature, concentration gradient will be formed on the diffusion contact surface with time [55]. This paper compares the change of its diffusion coefficient and summarizes the change rule of its diffusion behavior under different temperatures, different types of asphalts, and the addition of different rejuvenators. The mix ratio design of recycled asphalt mixture can be more accurate by analyzing the diffusion degree of the interface between old and new asphalt, so as to determine the construction process parameters. Thus, the utilization rate of recycled asphalt and the performance of recycled asphalt mixture can be improved.

## 4. Analysis of the Interface Fusion Effect of Old and New Asphalt

**Figure 5:**
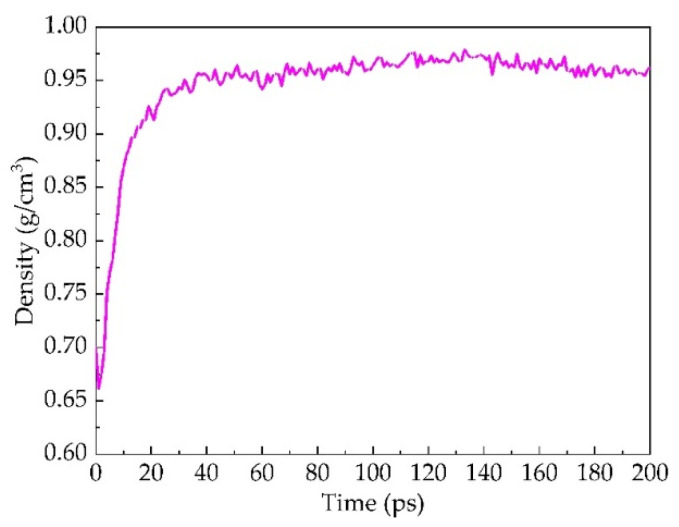
The dependence of density as a function of time for the old and new asphalt system.

As can be seen from Figure 5, taking the interface between Panjin 90# asphalt and aging Panjin 90# asphalt at 30 °C as an example, the density of asphalt increases sharply before 20 ps. After 20 ps, the density of asphalt converges to 1 g/cm^3^, indicating that the diffusion of asphalt density is basically complete.

### 4.1. Diffusion Process Analysis

**Figure 6:**
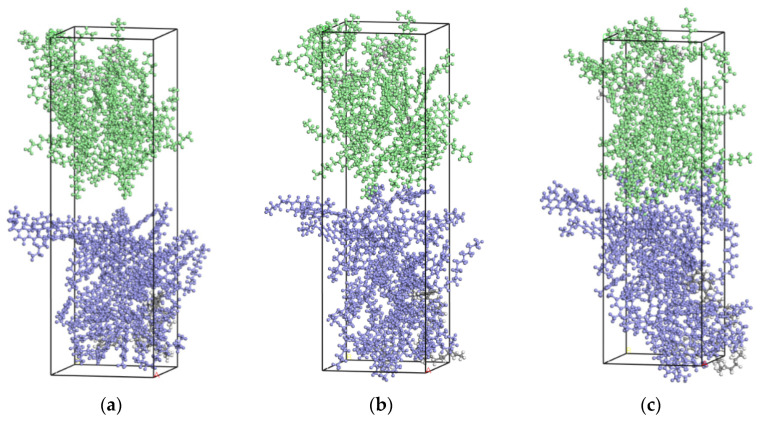
The new-old asphalt interface model diffusion process: (**a**) t = 0 ps; (**b**) t = 3 ps and (**c**) t = 20 ps.

Some researchers at the Institute of Technology found that the *MSD* rose rapidly at the beginning of the simulation and then gradually leveled off when using LAMMPS software (Version 2020, Sandia Corporation, Philadelphia, PA, USA), for the reason that the two systems quickly approached each other at the beginning of the simulation to fill in the gaps between them [43]. During this process, the rapid approach of molecules in the two systems resulted in a rapid increase in *MSD*. When the gap between the two systems is filled, the diffusion continues under the drive of intermolecular forces, and then the *MSD* gradually tends to be stable. It can be seen from the *MSD* diagram that the old and new asphalt starts to diffuse stably after 20 ps, while the curve of 20–150 ps is stable, and its fitting effect is the best. The simulation time was 200 ps due to the similar diffusion process at different temperatures. Taking Panjin 90# asphalt at a temperature of 293.15 K as an example, the process of new and old Panjin 90# asphalt stratification from close to each other to contact is shown in Figure 6.

After contact, the two systems will continue to diffuse due to the intermolecular van der Waals forces, however at a slower diffusion rate. As can be seen from Figure 6, only the part in contact with the surface has completed the fusion.

### 4.2. Diffusion of New Asphalt to Old Asphalt

**Figure 7:**
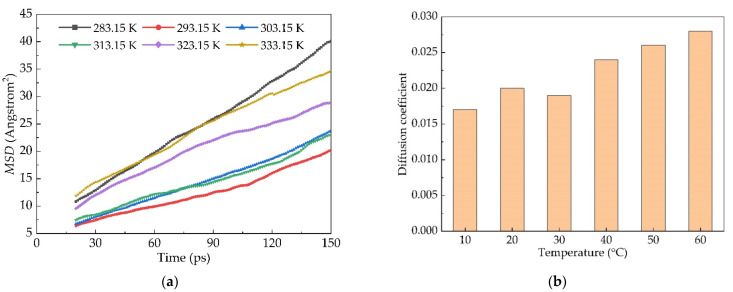
*MSD* and diffusion coefficient of Panjin 90# asphalt from new asphalt to old asphalt: (**a**) the dependence of *MSD* as a function of time and (**b**) the dependence of diffusion coefficient as a function of temperature.

The interface models with various simulation temperatures were established respectively by taking Panjin 90# asphalt as an example in order to study the degree of diffusion between new and old asphalt to each other and the influence of temperature on its diffusion. The pressure was 0.0001 GPa (one standard atmosphere), the force field was COMPASS, and 200 ps was simulated under the NPT ensemble. The stationary section of the *MSD* curve was selected to calculate the diffusion coefficient, and the results are shown as follows (in Figure 7):

The stable section of the *MSD* curve was selected for analysis according to the *MSD* change curve. Since the new and old asphalt were been in contact with each other after 20 ps, the section between 20 ps and 150 ps was selected and Origin software was used to perform linear fitting on the *MSD* image. It can be seen that with the increase in temperature, the diffusion coefficients show an upward trend. The diffusion coefficient increases by 10.6% with the temperature increasing by 10 °C through linear fitting.

### 4.3. Diffusion of Old Asphalt to New Asphalt

**Figure 8:**
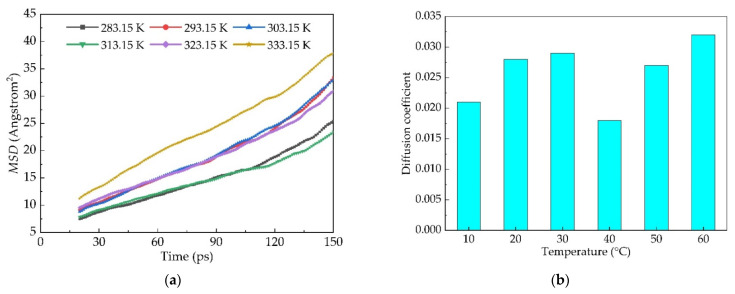
*MSD* and diffusion coefficient of Panjin 90# asphalt from old asphalt to new asphalt: (**a**) dependence of *MSD* as a function of time and (**b**) the dependence of diffusion coefficient as a function of temperature.

Considering the sharp rise in the first 20 ps and the final curve being slightly upward, according to the *MSD* curve, 20 ps~150 ps sections were selected and Origin software was used for linear fitting of the *MSD* images. As the temperature rises, the diffusion of old asphalt to new asphalt also shows an upward trend. The diffusion coefficient of old asphalt to new asphalt increases by 10.5% with the increase in temperature at 10 °C by linear fitting, as shown in Figure 8.

### 4.4. Comparison of Diffusion between Two Asphalt Types

**Figure 9:**
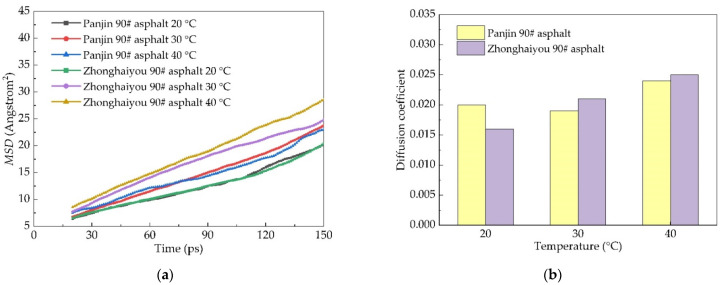
Comparison diagram of two asphalt *MSD* diagram and diffusion coefficient: (**a**) the dependence of *MSD* as a function of time and (**b**) the dependence of diffusion coefficient as a function of temperature.

The temperature was selected as 20 °C, 30 °C, and 40 °C, and other parameters remained unchanged to study the mean directional shift of the two kinds of asphalt in order to compare the diffusion behavior of Panjin 90# asphalt and Zhonghaiyou 90# asphalt, as shown in Figure 9.

Zhonghaiyou 90# asphalt is more sensitive to the influence of temperature compared with Panjin 90# asphalt, whose rising trend is more obvious with the increase of temperature.

### 4.5. Influence of Different Rejuvenators and Dosage on Diffusion

**Figure 10:**
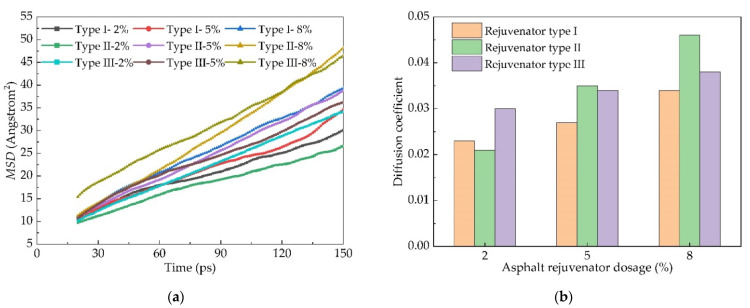
*MSD* of different rejuvenators and their diffusion coefficient: (**a**) the dependence of *MSD* as a function of time and (**b**) the dependence of diffusion coefficient as a function of rejuvenator dosage.

In order to explore the influence of different dosages of rejuvenator on diffusion, a model of a 90# asphalt with 2%, 5% and 8% of rejuvenators type I, type II and type III at 60 °C was established, respectively. Three kinds of mixtures with the different types of new asphalt rejuvenator and aging asphalt were then simulated by 200 ps molecular dynamics. The resulting *MSD* diagram is shown in Figure 10.

With the increase in dosage of the rejuvenator, the diffusion coefficient of the rejuvenator all showed an upward trend, among which the diffusion effect of rejuvenator type II on Panjin 90# asphalt was the most significant, and it was most affected by the content of the rejuvenator. Compared with Panjin 90# asphalt without a rejuvenator, its diffusion coefficient increases when the dosage is more than 5%, and the effect of rejuvenator type II is the most obvious. When the content of rejuvenator type II is 5%, its diffusion coefficient increases by 25%, while the diffusion coefficient increases by 64.3% when the content of rejuvenator type II is 8%.

## 5. Application Prospect

Research on reclaimed pavement has developed rapidly in China in recent years. From the initial thermal rejuvenation to the current cold recycling technology, the main purpose is to save energy and reduce the pollution caused by vaporized gas at high temperatures. As asphalt material is a typical temperature-sensitive material, whose flow performance usually reaches a better state at 160 °C, the cold recycling technology must overcome the difficulty of temperature. The performance of cold recycled mixtures is worse than that of hot rejuvenated mixtures, so the performance of cold recycled mixtures needs to be further improved.

According to the above analysis of the new and old asphalt diffusion mixing cold rejuvenation mode, the surface diffusion and miscibility degree of the new and old asphalt rejuvenation and RAP is limited, far from reaching 100% fusion, and the inner layer is coated with old RAP asphalt, which does not participate in the rejuvenation, leading to different surface coating thickness of the new and old aggregates. This will inevitably affect the performance of the recycled asphalt mixture, and the diffusion characteristics of old and new asphalt has an important effect on the rejuvenation effect of aging asphalt, which also explains why most cold recycled mixtures are prone to lack of strength in the early stage after normal pavement installation and fail to achieve the desired effect [56].

Temperature, type of asphalt, and type of rejuvenator all have significant effects on the degree of diffusion. Therefore, the mixing degree of old and new asphalt cannot be generalized. For RAP in different areas, the different degree of diffusion must be considered due to different mixture ratios, asphalt type, and modifier. How to ensure that the interface fusion effect can achieve the desired effect is also a key problem in different temperatures, especially in cold areas. As for selection of the rejuvenator, it is more expensive than that of asphalt, so a rejuvenator with higher efficiency and lower price should be sought. The rejuvenation mechanism supplements the components lost by old asphalt; thus, bio-oil with relatively low price can be considered. The influence law of diffusion degree on the performance of recycled asphalt mixture can be further explored after clarifying the diffusion law and the fusion effect of the old asphalt interface, so as to make the mixing ratio of the asphalt mixture more reasonable, providing guidance for the optimization of construction technology and improving the utilization rate of RAP used asphalt, thus improving the performance of the recycled asphalt mixture.

## 6. Conclusions

(1) The fusion degree of the asphalt interface conforms to the partial fusion theory. It can be seen that only a small part of the contact between the two interfaces is fused from the diffusion process of the interface model.

(2) Temperature has a significant effect on its diffusion behavior in the diffusion process of new-old asphalt. The diffusion coefficients of new asphalt to old asphalt and old asphalt to new asphalt are similar, according to linear fitting. The degree of diffusion of new asphalt to old asphalt and the degree of diffusion of old asphalt to new asphalt changed with temperature.

(3) The diffusion coefficient of Zhonghaiyou 90# asphalt is smaller than that of Panjin 90# asphalt when the temperature is 20 °C; the situation is opposite when the temperature is higher than 20 °C.

(4) There are two main processes for the diffusion of old and new asphalt. First, at the beginning of the diffusion, the new and old asphalt systems quickly move towards each other to fill the gap. The *MSD* increased significantly in this stage, the diffusion coefficient was greater than that in the second stage, and the time was short, which is basically completed within 20 ps. Secondly, self-diffusion occurs after new and old asphalt contact under the action of intermolecular van der Waals forces, and the growth rate of *MSD* at this time is relatively stable and slow.

(5) Among the three rejuvenators, rejuvenator type II has the most significant effect on its diffusion. When the dosage is 8%, the diffusion coefficient of Panjin 90# asphalt increases by 64.3% compared with that of Panjin 90# asphalt without a rejuvenator.

## Figures and Tables

**Table 1:** Composition of all asphalt components.

Asphalt Type	Asphaltenes (%)	Naphthene Aromatics (%)	Polar Aromatics (%)	Saturates (%)
Panjin 90# asphalt	6.47	33.57	28.07	31.89
Aging Panjin 90# asphalt	14.96	38.83	18.55	27.66
Zhonghaiyou 90# asphalt	5.07	45.17	31.55	18.21
Aging Zhonghaiyou 90# asphalt	12.08	35.89	30.32	21.70

**Table 2:** Solubility of various asphalt components (J/cm^3^)^1/2^.

Asphalt Type	Asphaltenes	Naphthene Aromatics	Polar Aromatics	Saturates
Panjin 90# asphalt	15.31	16.36	18.51	14.57
Zhonghaiyou 90# asphalt	14.20	17.58	18.51	14.57

## Data Availability

The data presented in this study are available on request from the corresponding author.
